# Derivation and Validation of a Necroptosis-Related lncRNA Signature in Patients with Ovarian Cancer

**DOI:** 10.1155/2022/6228846

**Published:** 2022-05-23

**Authors:** Linling Zhu, Jiaoyan He, Xinyun Yang, Jianfeng Zheng, Wenhua Liu, Hao Chen

**Affiliations:** ^1^Department of Pathology, Hangzhou Women's Hospital, Hangzhou, China; ^2^Department of Gynecology, Hangzhou Women's Hospital, Hangzhou, China; ^3^Department of Gynecology, Zhuji People's Hospital of Zhejiang, Shaoxing, China; ^4^Department of Reproductive Endocrinology, Women's Hospital, School of Medicine, Zhejiang University, Hangzhou, China

## Abstract

**Background:**

Ovarian cancer (OC) is the leading cause of gynecologic malignant tumors. The role of necroptosis-related lncRNAs (NRLs) in OC remains unclear. This study aims to explore the association between NRLs and prognosis in OC patients.

**Methods:**

The Cancer Genome Atlas (TCGA) and GTEx datasets were used to obtain OC's data. A NRLs signature associated with overall survival (OS) was constructed by Cox-LASSO regression analysis in training cohort for calculating risk score and then validated in testing cohort. Subsequently, the area under the curve (AUC) and Kaplan–Meier survival analysis were used to evaluate the predictive accuracy of the risk score. Finally, the immune infiltration and functional enrichment were compared between different risk groups.

**Results:**

A 8-NRLs signature including AC245128.3, AL355488.1, AC092794.1, AC068888.2, AL590652.1, AC008982.2, FOXP4-AS1, and Z94721.1 was identified to assess the OS of OC. Kaplan–Meier survival analysis, AUC value, and Cox regression analysis confirmed its predictive value and showed that the clinical outcomes were worse for high-risk patients. There were also differences in immunological functioning and immune pathways between the high-risk and low-risk groups.

**Conclusions:**

The signature based on eight NRLs has significant values in predicting prognostic prediction in OC, as well as providing a new sight for targeted therapies.

## 1. Introduction

Ovarian cancer (OC) is a common cancer which is the leading cause of cancer-related mortality in the reproductive system worldwide. In Asian countries, its incidence showed an upgraded trend in recent years [1]. As a heterogenous group of malignancies, its prognosis is obviously different in various pathological stages and classifications. Despite advances in early detection and drug development, clinical outcomes still remain unsatisfactory. When diagnosed, 60%–75% of patients are already in the middle and advanced stages (stage III-IV) and their 5-year overall survival (OS) rate is less than half [2]. There is an urgent need to search for accurate predictors and prospective prognostic markers in order to contribute to a better prognosis of OC and give trustworthy information to guide suitable personalized treatment options.

Necroptosis is a type of planned cell death that combines the characteristics of both necrosis and apoptosis [3]. Recent researches have revealed the important role of necroptosis in tumorigenesis and metastasis [4]. Meanwhile, as a member of the noncoding RNA family, long noncoding RNA (lncRNA) lacks the capability of encoding a protein [5]. Along with the development of related researches, accumulating studies suggest that lncRNAs act as a vital role in development, progression, cell survival, and genesis of tumor [6, 7]. Many lncRNAs are associated with drug response in various cancers, including OC [8]. To date, emerging evidence has shown the potential of lncRNAs in regulating necroptosis for cancer biology [9]. However, the clinical significance of necroptosis-related lncRNAs (NRLs) remains largely unknown. Remarkably, it is crucial to figure relevant lncRNAs closely linked to necroptosis and prognosis in OC.

This research was conducted to analyze the lncRNAs expression dataset in OC from GTEx database and The Cancer Genome Atlas (TCGA), and screened for NRLs with prognostic value. An eight autophagy-related lncRNA signature of predicting the OC prognosis was identified.

## 2. Materials and Methods

### 2.1. Datasets and Data Preprocessing

The TCGA and GTEx databases were used to gather the clinical data (*n* = 587), RNA sequencing profiles (*n* = 379), and normal ovarian epithelial tissue RNA sequencing profiles (*n* = 88) for OC. Only 374 individuals were kept for further study after excluding patients lacking RNA sequencing and survival time. The patients were split into two groups (training cohort and testing cohort) at a ratio of 3 : 7 using the *R* software “caret” package. With the usage of annotation documents of the GENCODE database, lncRNAs and protein-coding genes were identified. Moreover, based on earlier research, 67 necroptosis-related genes (NRGs) were retrieved. Meanwhile, the “limma” program was used to investigate the differences in NRG expression between OC and normal samples. Subsequently, using the 13,832 lncRNAs and differential expression NRGs discovered, Pearson correlation analysis was performed (*p* < 0.001, correlation coefficient >0.4). A total of 161 NRLs were eventually chosen for further bioinformatics investigation.

### 2.2. Construction of a Prognostic Signature

Univariate Cox regression analysis was conducted to identify prognostic lncRNAs (*p* value <0.05). These predictive lncRNAs were then used to identify lncRNAs implicated in signature creation with multivariate Cox, least absolute shrinkage and selection operator (LASSO) regression analysis. To develop the model and manage the complexity of LASSO regression, we employed the suitable *λ*. The following formula was used to determine the risk score: risk score for

(1) $$\begin{array}{c} \text{OS}=\sum_{i=1}^{n}{\text{Coef}}_{i}*x_{i}. \end{array}$$

In addition, patients with different prognostic lncRNAs expressions were classified by using ConsensusClusterPlus package. By locating the inflection point of the sum of squared errors, the ideal *k* value was determined (SSE). The decline slowed down after *k* = *i*, and *k* = *i* was selected.

### 2.3. Exploring Clinical Benefit

The risk score for each OC patient was calculated using the algorithm above. Risk signatures for predicting survival were assessed by area under the curve (AUC) and Kaplan–Meier survival analysis. The median value in the ROC curve, which is used to choose “high-risk” and “low-risk” groups, was determined by calculating the risk score of each patient. In training and testing cohorts, we pooled clinical variables and ran univariate and multivariate Cox regression analyses, respectively. Finally, based on the coefficients of the above multivariate Cox regression, to create a nomogram, we utilized *R* software “regplot” package.

### 2.4. Immune Infiltration Analysis

We employed 6 methods (TIMER, CIBERSORT, QUANTISEQ, MCP-counter, XCELL, and EPIC) to estimate the abundances of immune cells classified by risk groups in order to investigate variations in immune cell infiltration. SsGSEA and ESTIMATE algorithms were also performed to compare immunological functions and pathways in low- and high-risk groups. More crucially, we looked at how immunological checkpoint and human leukocyte antigen-associated genes were expressed in various risk groups. The “ggplot2” and “clusterProfiler” programs in *R* software were then used to conduct gene GSEA enrichment analysis using differently risk groupings.

### 2.5. Drug Sensitivity Analysis

The IC50 was computed in *R* using the pRRophetic package, and the medicines were found in the Genomics of Drug Sensitivity in Cancer database.

### 2.6. Statistical Analysis

The R programming language conducted all statistical analyses (v.4.0.1). In the preceding section, detailed statistical approaches for transcriptome data processing were discussed. The difference is statistically significant when *P* < 0.05.

## 3. Results

### 3.1. Landscape of Necroptosis-Related Genes

**Figure 1:**
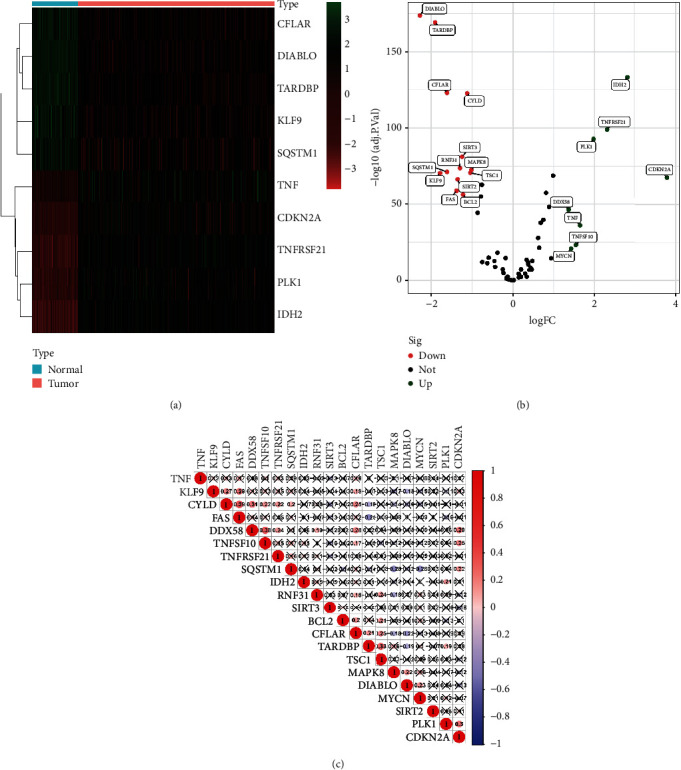
Identification of necroptosis-related genes in ovarian cancer. (a) The heatmap of the differentially expressed necroptosis-related genes. (b) The volcano plot of necroptosis-related genes with differential expression. (c) A heatmap for correlation analysis of 21 necroptosis-related gene expressions in ovarian cancer tissues.

According to the expression of 67 genes associated with necroptosis between normal and tumor samples, we finally got 21 specific necroptosis-related genes NRGs in OC (|Log2FC|>1 and *P* < 0.05), as shown in Figure 1(a). Of them, 8 were upregulated, and 13 were downregulated (Figure 1(b)). As shown in Figure 1(c), the correlation analysis of 21 NRGs showed that DDX58 had the strongest positive correlation with CYLD (*r* = 0.39).

### 3.2. Identification of NRLs

**Figure 2:**
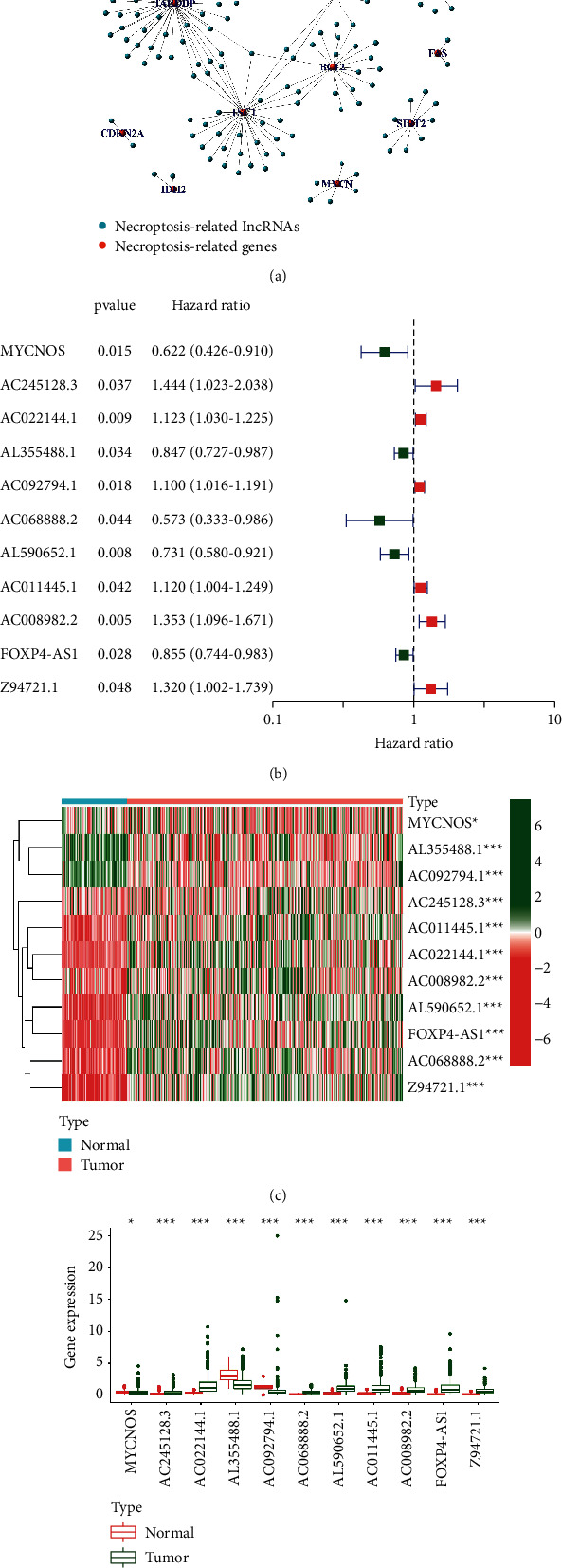
Identification of necroptosis-related lncRNAs in ovarian cancer. (a) The network necroptosis genes and lncRNAs interact in. (b) The HR and *p* value of 11 prognostic lncRNAs from the univariable Cox HR regression. (c) The expression profiles of 11 prognostic lncRNAs. (d) The boxplot of 11 prognostic lncRNAs.

For TCGA-OC cohort, the aforementioned 21 NRGs, as well as all annotated lncRNAs, were used and analyzed through Pearson correlation analysis (correlation coefficients>0.4 and *p* < 0.001). 161 NRLs were identified (Figure 2(a)). These NRLs were adopted in univariate Cox analysis to find survival-related NRLs. Finally, for the following analyses, 11 NRLs were examined (Figure 2(b)). Interestingly, the Wilcox test revealed that all survival-related NRLs were highly expressed in the tumors except MYCNOS and AC245128.3, which was highly expressed in the normal samples (Figures 2(c)-2(d)).

### 3.3. Construction of a Risk Signature

**Figure 3:**
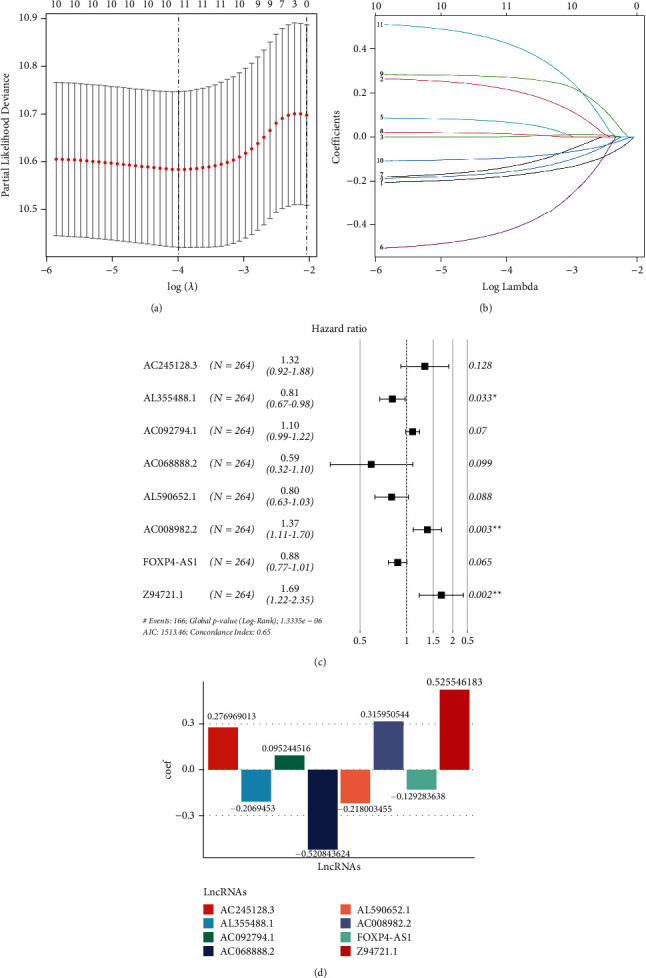
Construction of necroptosis-related lncRNAs prognostic signature in ovarian cancer. (a) The optimal values of the penalty parameter were determined by 10-round cross-validation. (b) The LASSO Cox analysis showed eight lncRNAs most closely linked to prognosis. (c) Univariate Cox regression analysis was used to derive prognostic lncRNAs. (d) Coefficient of each lncRNA in multivariate Cox regression analysis.

A total of 11 NRLs were subjected to LASSO regression analysis in order to reduce the number of genes in the signature (Figures 3(a)-3(b)). After that, 10 NRLs were recovered using LASSO and submitted to multivariate Cox regression analysis (stepwise method) to develop a risk stratification system (Figure 3(c)). Eventually, the risk score for OC patients was determined by multiplying the expression of 8-NRLs by the regression coefficients: risk score = (0.2769 ^*∗*^ AC245128.3) + (−0.2069 ^*∗*^ AL355488.1) + (0.0952 ^*∗*^ AC092794.1) + (0.5208 ^*∗*^ AC068888.2) + (−0.2180 ^*∗*^ AL590652.1) + (0.3159 ^*∗*^ AC008982.2) + (−0.1292 ^*∗*^ FOXP4-AS1) + (0.5255 ^*∗*^ Z94721.1) (Figure 3(d)).

### 3.4. Clinical Benefits of Risk Signature

**Figure 4:**
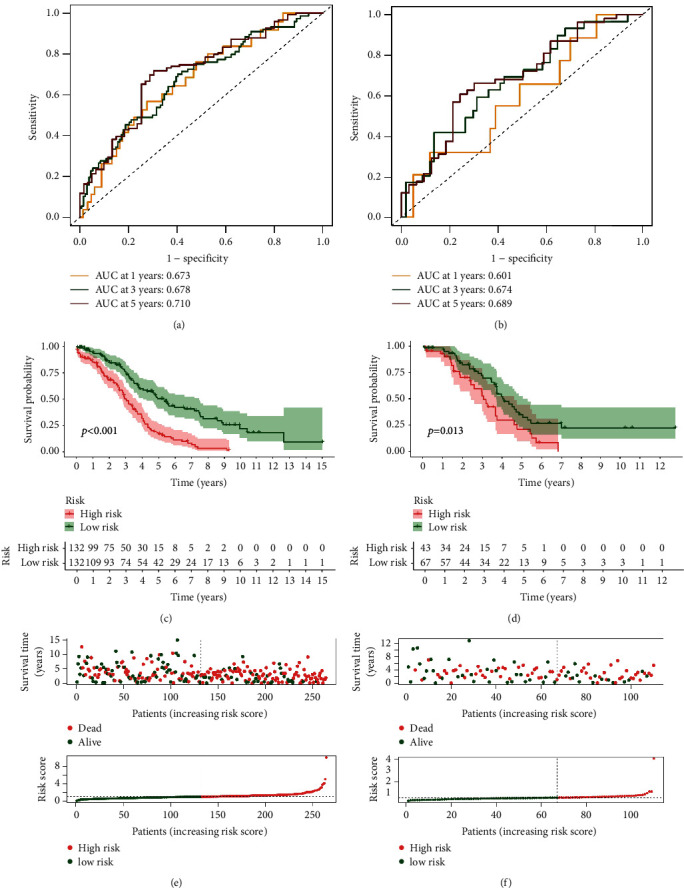
The predictive value of the prognostic model in both the training and test cohorts. Time-dependent ROC curve of ovarian cancer patients in the training cohort (a) and test cohort (b). Kaplan–Meier curves of ovarian cancer patients in the testing cohort (c) and test cohort (d). The scatter plot of the sample survival overview and the risk curve based on the risk score in the testing cohort (e) and test cohort (f), respectively.

**Figure 5:**
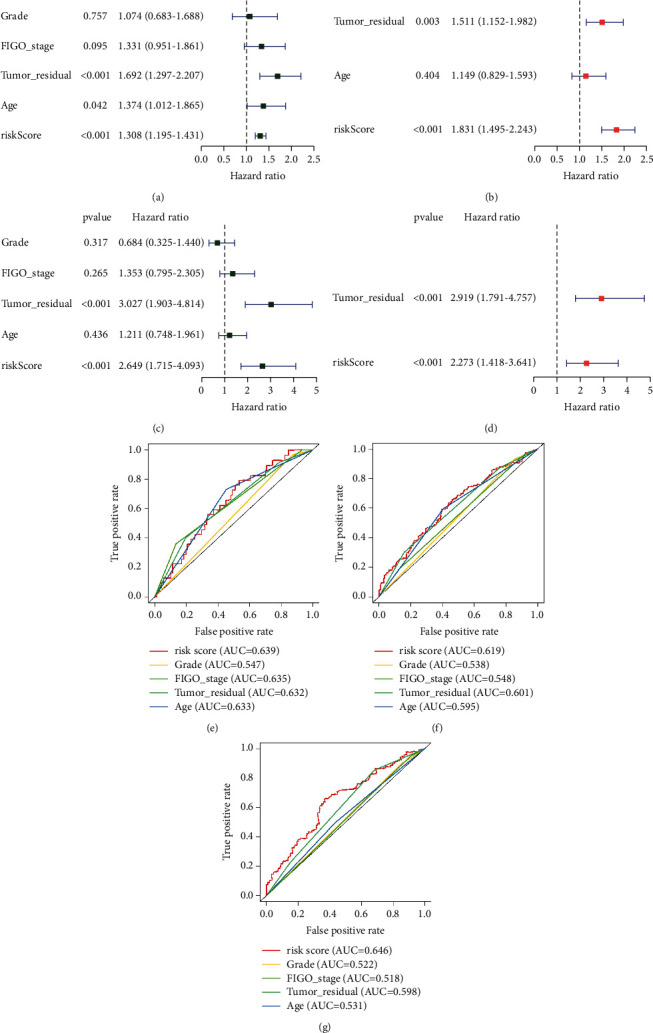
Assessment of the independent prognostic risk model of the 8 necroptosis-related lncRNAs in ovarian cancer. Univariate Cox analyses of clinical factors and risk score with OS in the training cohort (a) and test cohort (b). Multivariate Cox analyses of clinical factors and risk score with OS in the training cohort (c) and test cohort (d). Calculate the AUC for risk score, grade, FIGO stage, tumor residual, and age of the total survival risk score according to the ROC curve for 1-, 3-, and 5-year OS (e–g). OS, overall survival.

The OC patients of TCGA cohort were separated into two risk categories based on the median value of risk scores: high risk and low risk. The AUCs of risk score computed with the training cohort at 1, 3, and 5 years were 0.673, 0.678, and 0.710, respectively (Figure 4(a)). Meanwhile, the AUC for predicting 1, 3, and 5 years was 0.601, 0.674, and 0.689, respectively, in testing cohort (Figure 4(b)). In each group, high-risk patients had a considerably lower survival time than low-risk ones (*p* < 0.05), as shown in Figures 4(c)-4(d). In addition, the distribution of risk score, survival status, and survival time was compared between low- and high-risk groups in the training and testing cohorts using the risk score algorithm. All of these showed that the high-risk group had a poor prognosis (Figures 4(e)-4(f)). Univariate and multivariate Cox regression analyses employing clinical characteristics and risk score were used to determine if the risk score was an independent prognostic variable for OC patients. The risk score was strongly associated with OS in both the training and testing populations, according to the results of univariate Cox regression analysis (training cohort: HR = 1.308, 95% CI = 1.195–1.431; testing cohort: HR = 2.649, 95% CI 1.715–4.093) (Figures 5(a), 5(c)). In multivariate Cox regression analysis, the risk score remained an independent marker for OS after correcting for other covariates (training cohort: HR = 1831, 95% CI = 1.495–2.243; testing cohort: HR = 2.273, 95% CI = 1.418–3.641) (Figures 5(b), 5(d)). In addition, the risk mark outperformed the other clinicopathological markers in terms of prediction, according to ROC curve analysis (Figures 5(e)–5(g)).

### 3.5. Construction of Nomogram

**Figure 6:**
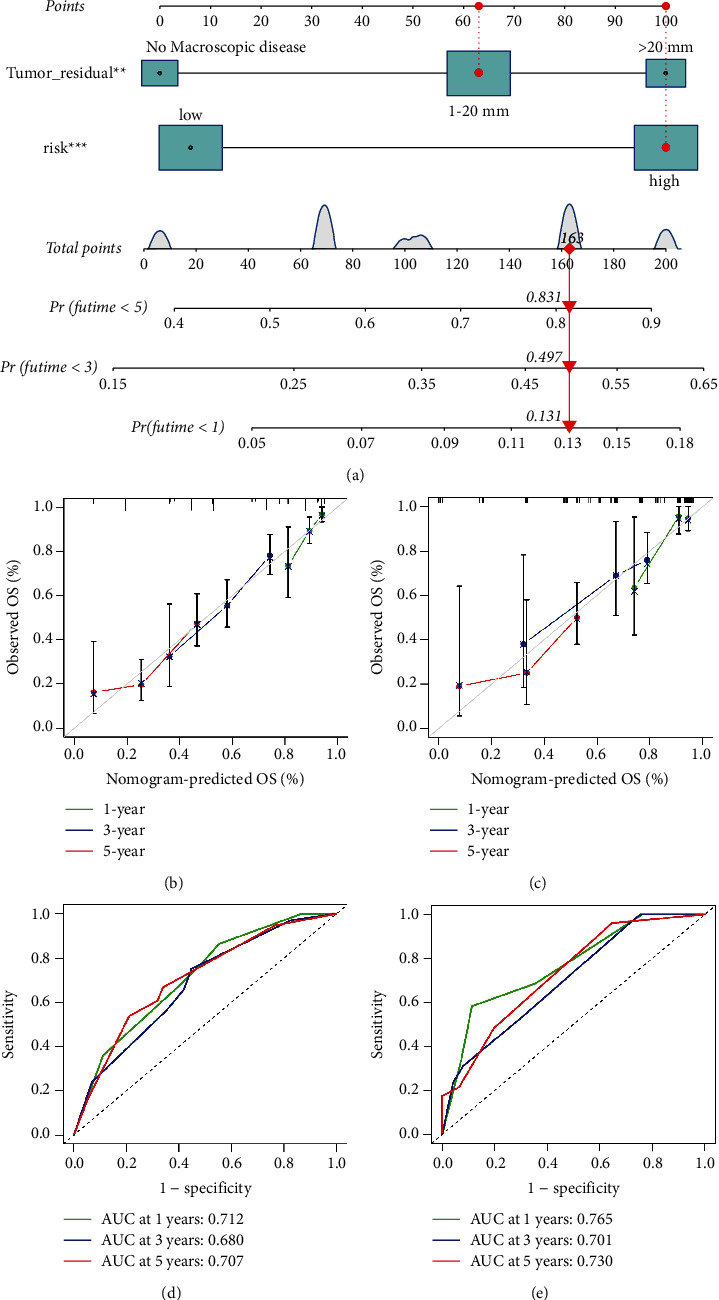
Construction and evaluation of the signature-based prognostic nomogram predicting OS. (a) The prognostic nomogram for the prediction of 1-, 3- and 5-year OS in ovarian cancer. The calibration plots of the testing cohort (b) and test cohort (c). The ROC of the nomogram for predicting 1-, 3-, and 5-year OS in the testing cohort (d) and test cohort (e). OS, overall survival.

We depicted the risk signature based on the aforementioned risk formula since the risk signature's formula is hard and the nomogram may intuitively relate to clinical practice. In multivariate Cox regression, we merged statistically significant indications to create a visual prognostic model (Figure 6(a)). Furthermore, the nomogram's calibration curve revealed that the prediction curves in two cohorts are almost identical to the standard curve. The projected survival rate is closely similar to the actual rates at 1, 3, and 5 years, as illustrated in Figures 6(b)-6(c). Furthermore, according to the ROC analysis of nomogram, good predictive performance for the training and testing cohorts was revealed (Figures 6(d)-6(e)).

### 3.6. Immunity Analysis

**Figure 7:**
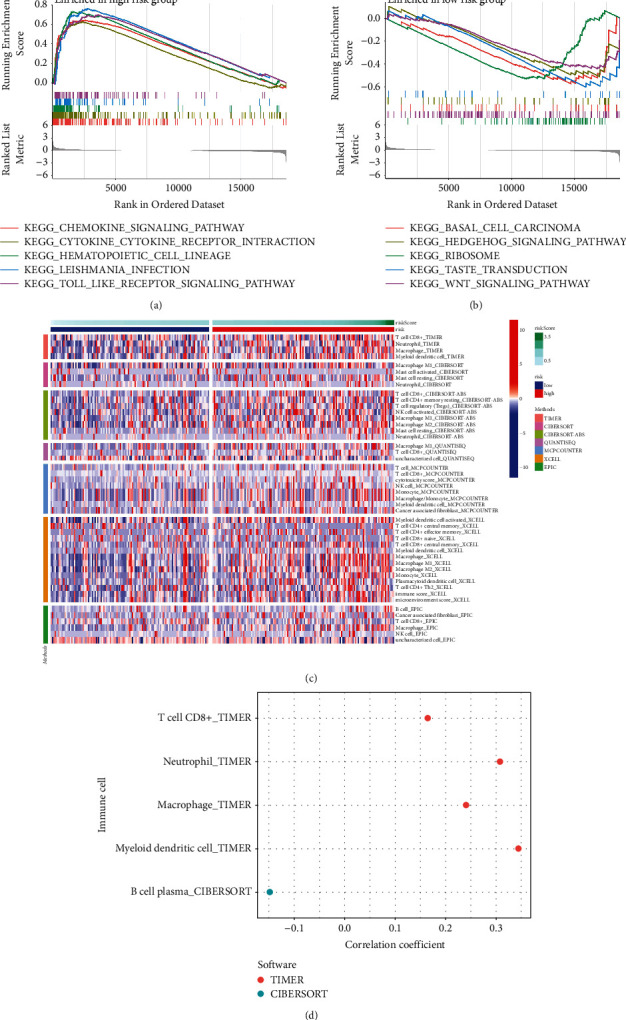
GSEA between high- and low-risk groups. GSEA of the top 5 pathways significantly enriched in the high-risk group (a) and low-risk group (b). (c) 6 immune algorithms to estimate the abundances of immune cells in various risk groups. (d) The correlation between different immune cells and the risk score. GSEA, gene set enrichment analysis.

**Figure 8:**
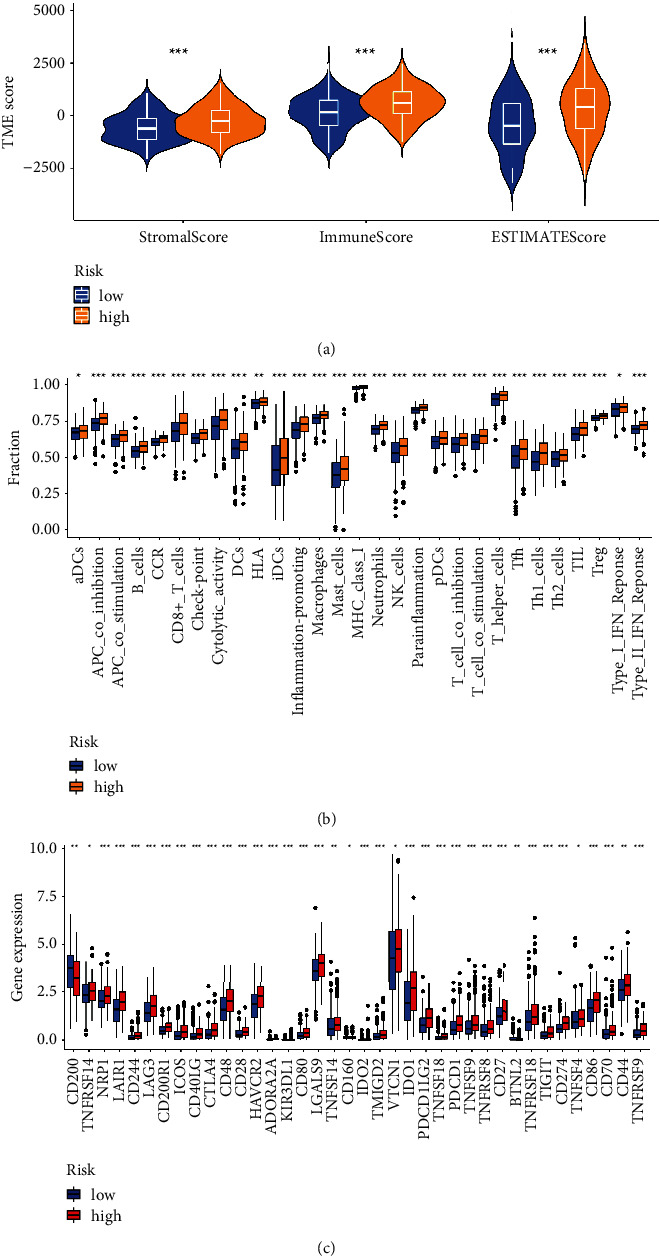
The immune status difference between the low- and high-risk ovarian cancer patients. (a) Violin plots of tumor purity for the low- and high-risk groups. (b) The low-risk and high-risk groups were compared in terms of immune-related functioning. (c) The expression of checkpoints differs among risk groups.

The main enrichment pathways of various risk categories were investigated using the GSEA algorithm. In the high-risk group, CHEMOKINE SIGNALING PATHWAY, CYTOKINE RECEPTOR INTERACTION, and HEMATOPOIETIC CELL LINEAGE were dominant (Figure 7(a)). In the low-risk group, important pathways were BASAL CELL CARCINOMA, HEDGEHOG SIGNALING PATHWAY, and RIBOSOME (Figure 7(b)). A heatmap of immune infiltration was created, based on six algorithms (TIMER, CIBERSORT, QUANTISEQ, MCP-counter, XCELL, and EPIC) (Figure 7(c)) to investigate the association between various risk groups and immune cell infiltration. Interestingly, in TIMER algorithm, T cell CD8^+^, neutrophil, macrophage, and myeloid dendritic cell were positively correlated with risk score (Figure 7(d)). Meanwhile, results of ESTIMATE algorithm revealed high-risk group had a higher score in stromal, immune, and estimate (Figure 8(a)). Those who are at high risk are more likely to develop hot tumors and react to immunotherapy. The “ssGSEA” *R* package was used to quantify the enrichment scores of immune cell subpopulations and their related activities in order to further investigate the relationships between risk scores and immune cells and functions (Figure 8(b)). Given the significance of checkpoint immunotherapy, it is worth emphasizing that there are large variances in immune checkpoint expression across various risk categories (Figure 8(c)).

### 3.7. Drug Effectiveness Analysis

**Figure 9:**
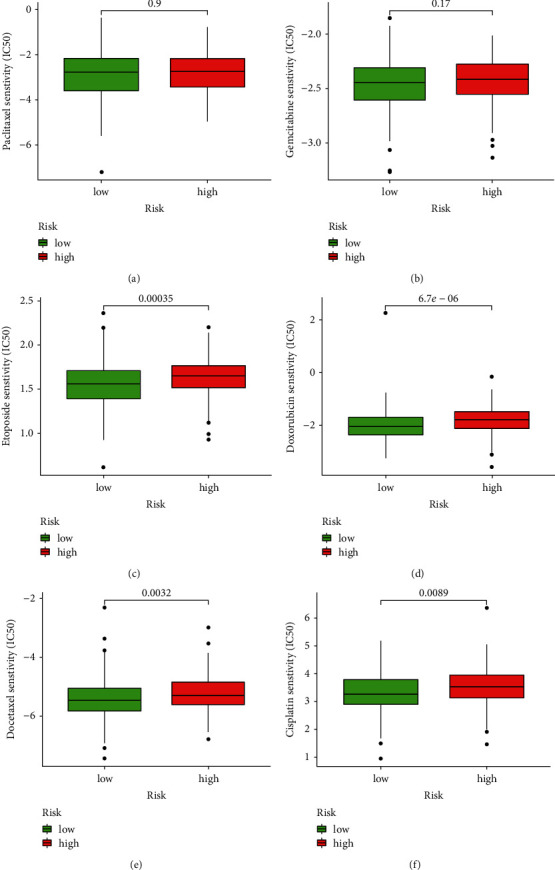
The sensitivity of chemotherapeutic medicines in different risk groups. (a) Paclitaxel. (b) Gemcitabine. (c) Etoposide. (d) Docetaxel. (e) Doxorubicin. (f) Cisplatin.

The drug sensitivity of OC chemotherapeutic drugs, which are often used in clinics, was examined. Six chemotherapeutic drugs had their IC50 values measured in OC patients, and four of them had statistically significant differences across risk subgroups (Figure 9). The high-risk group had considerably higher IC50 values of etoposide, docetaxel, doxorubicin, and cisplatin (*P* < 0.05). It was discovered that OC patients in the low-risk group, as determined by their risk profile, were more responsive to the chemotherapeutics mentioned above.

### 3.8. Molecular Subtypes Based on NRLs

**Figure 10:**
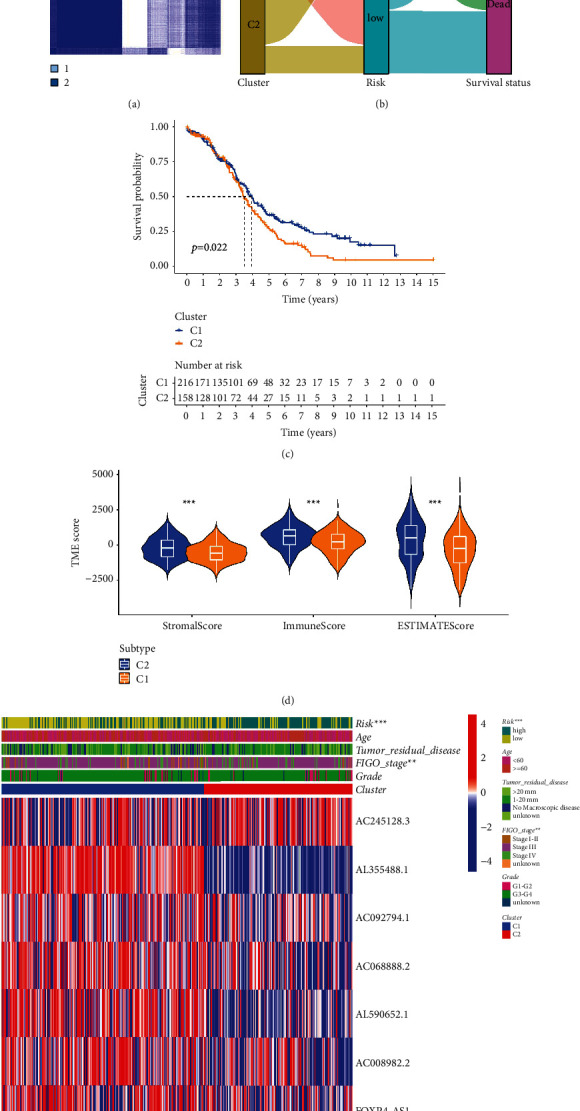
The molecular subtypes based on lncRNAs linked to necroptosis. (a) Heatmap of optimal k value by clustering algorithm. (b) Association between subtypes and risk groups. (c) Kaplan–Meier curves in the different subtypes. (d) TME scores in the different subtypes. (e) Heatmap of clinical correlation combined with clinical and pathological factors.

The OC patients from TCGA were divided into different subtypes based on consensus algorithm and 8 NRLs expression in risk signature (Figure 10(a)). Interestingly, the majority of C2 subtypes were classified as high-risk group, and the majority of C1 subtypes belonged to the low-risk group (Figure 10(b)). C2 showed the worst prognostic efficacy, while C1 had a higher probability of survival (Figure 10(c)). Moreover, results of ESTIMATE algorithm revealed C2 group had a higher score in stromal, immune, and estimate. Heatmap also showed that the molecular subtypes were statistically significant with risk groups and FIGO staging (Figure 10(d)). Taken together, our molecular subtype results provided another insight into the ability of 8 NRLs to differentiate patients (Figure 10(e)).

## 4. Discussion

OC is a heterogenous disease. Although overall survival rates for OC patients have improved greatly, metastasis and recurrence are the leading causes of death [10]. Age, pathological stage, lymph node metastasis, and distant metastasis have all been employed in clinical studies to predict the prognosis of OC, but the accuracy is inadequate. The different clinical outcomes in patients with the same staging indicate that the traditional staging system is not fully functional to predict the prognosis. Relevant biomarker tumor of prognosis needs to be investigated urgently. Many aspects of ovarian cancer biology are regulated by lncRNAs [11]. Necroptosis is a sort of cell death which is regulated, acting as a double-edged sword in the cancer development. Key mediators of its pathway alone or in combination can promote tumor metastasis and progression [12]; however, necroptosis, on the other hand, has also been reported as a fail-safe mechanism that protects against tumor development when apoptosis is impaired [13]. Most certainly, necroptosis plays a big part in cancer. The lncRNAs related to necroptosis may be novel molecular biomarkers and therapeutic targets for OC. Owing to the important role of necroptosis in cancer, its related lncRNA has aroused more attention. With unprecedented accumulation of tumor data in international public databases, now is the new era of data technology for OC research [14, 15].

lncRNAs were involved in development, progression, and metastasis of cancer through various signal pathways [16, 17]. Recent report introduced a prognostic risk model of NRLs in gastric cancer [18], while the significance of NRLs in OC prognosis was yet unknown. Twenty-one specific NRGs were differentially expressed between normal and OC samples. Based on it, we identified the 8 lncRNA prognostic signatures related to necroptosis (AC245128.3, AL355488.1, AC092794.1, AC068888.2, AL590652.1, AC008982.2, FOXP4-AS1, and Z94721.1) as an adjunct to established clinical prognostic factors for OC. Until now, a number of studies of FOXP4-AS1 (lncRNA forkhead box P4 antisense RNA 1) in cancer had been reported. An experimental study demonstrated that by sequestering miR-3184-5p to upregulate FOXP4-AS1, high expression of FOXP4-AS1 boosted cell proliferation and inhibited apoptosis, demonstrating an oncogenic effect in prostate cancer [19]. Zhao et al. [20] found FOXP4-AS1 had a significant influence in cervical cancer development. Additionally, FOXP4-AS1 acted as an unfavorable prognostic factor in neoplastic disease, such as nasopharyngeal carcinoma, mantle cell lymphoma, and colorectal cancer [21–23]. Also, AC092794.1 was used to construct a risk signature for lung adenocarcinoma [24]. So far, little is known regarding the effect of other lncRNAs. In consequence, it is necessary to conduct experiments to further clarify their biological functions in OC.

To explore the signature feasibility, univariate and multivariate COX analysis, and ROC analysis were applied to compare this prognostic feature with clinical indexes. The samples were separated into high-risk and low-risk subgroups based on the median risk score. High-risk individuals had a worse clinical result. The findings revealed that the NRL signature might be a predictive factor for OC patients, which was reliable and stable. It was also suggested that the risk model of the 8 NRLs is superior to other clinicopathological factors. The relevance of the NRL signature in OC was further shown by correlative immune algorithms. The immune cell enrichment analysis revealed a close connection between necroptosis and tumor immunity. The high-risk and low-risk groups had significantly different immune scores, stromal scores, and ESTIMATE scores. High-risk groups might be more prone to be hot tumors and responding to immunotherapy.

Precision medicine, focused on identifying prognosis-specific predictors of survival, will bring new therapeutic strategies, drug discovery, and gene-oriented treatment. This research, therefore, is under way. As well as predicting prognosis, the NRLs signature has the additional advantage of references of clinical rational administration. The newly acquired NRLs knowledge might aid us in gaining a better grasp of OC, which may follow by significant innovations and clinical solutions for treating cancers.

However, there are several deficiencies in this study. The study had a limited sample size; hence, the real-world data of multicenter clinical cohort are needed to validate the prognostic signature. In addition, due to the lack of researches on necroptosis in OC, its mechanism remains to be further explored through functional experiments. We are currently collecting clinical specimens and data in preparation for future study.

## 5. Conclusions

In summary, a novel necroptosis-related prognostic risk model consisting of 8 lncRNAs (AC245128.3, AL355488.1, AC092794.1, AC068888.2, AL590652.1, AC008982.2, FOXP4-AS1, and Z94721.1) was identified for OC patients. Furthermore, the lncRNA signature may be used to guide personalized treatment and enhance the prognosis of OC patients. Given the scarcity of studies on the mechanism and interactions among various NRLs in OC, further research is needed to confirm the clinical utility and reveal the underlying pathways.

## Data Availability

The data that support the findings of this study are available at the TCGA (https://tcga-data.nci.nih.gov/tcga/) and GTEx (https://www.gtexportal.org/).
